# Advantages of using molecular coancestry in the removal of introgressed genetic material

**DOI:** 10.1186/1297-9686-45-13

**Published:** 2013-05-01

**Authors:** Carmen Amador, Jesús Fernández, Theo HE Meuwissen

**Affiliations:** 1 INIA, Ctra, De La Coruña Km. 7.5, Madrid, 28040, Spain; 2 Institute of Animal and Aquacultural Sciences, Norwegian University of Life Sciences, Ås, N-1432, Norway

## Abstract

**Background:**

When introgression of undesired exogenous genetic material occurs in a population intended to remain pure, actions are necessary to recover the original background. It has been shown that genome-wide information can replace pedigree information for different objectives and is a valuable tool in the fields of genetic conservation and breeding. In this simulation study, molecular information provided by 50 000 SNP was used to minimise the molecular coancestry between individuals of an admixed population and the foreign individuals that originally introgressed a native population in order to remove the exogenous DNA.

**Results:**

This management method, which detects the ‘purest’ individuals to be used as parents for the next generation, allowed recovery of the native genetic background to a great extent in all simulated scenarios. However, it also caused an increase in inbreeding larger than expected because of the lower number of individuals selected as parents and the higher coancestry between them. In scenarios involving several introgression events the method was more efficient than in those involving a single introgression event because part of the genetic information was mixed with the native genetic material for a shorter period.

**Conclusions:**

Genome-wide information can be used to identify the purest individuals via the minimisation of molecular coancestry between individuals of the admixed and exogenous populations. Removal of the undesired genetic material is more efficient with a molecular-based approach than with a pedigree-based approach.

## Background

Depending on the situation, crossbreeding can be considered as a positive or negative process for the management of populations. Many studies have analysed the benefits of a new genetic input: gene flow between populations can offset the loss of genetic diversity and avoid the deleterious consequences of inbreeding [1,2]. However, exchange of genetic material can have disadvantages. Introgression can lead populations to extinction, a phenomenon more likely to occur today because the number of invasive species threatening wild populations has considerably increased due to human activities [3,4]. In the field of domestic animals, maintaining pure populations can be essential either to secure quality characteristics of livestock products [5] or to breed animals for other economic reasons such as horse breeds for competitions [6] and dog breeds for aesthetic reasons [7].

Livestock breeds are important components of the world’s biodiversity [8]. Local breeds have been selected to fit a wide range of environmental conditions and human needs. The genetic diversity of livestock breeds represents a reservoir to select for new characteristics in response to changes in environment, to diseases, or to new demands in food quality or quantity. Intensive selection of a few highly productive breeds has caused the decline of numerous other breeds that often possess special adaptive characteristics (to harsh conditions, disease resistance, etc.) not found in the former [9,10]. In many cases, crossbreeding between a local breed and a more productive breed leads to the disappearance of the specific features and adaptive traits of the local breed. Therefore, they should be recovered to avoid population extinction [11-13].

In a previous study, we used pedigree data as source of information to remove introgressed genetic material and recover the native genetic background [14]. Different introgression events were simulated with a varying number of exogenous individuals entering the population and different numbers of generations during which exogenous genetic material was admixed. Based on information from a completely recorded genealogy, this method optimised the genetic contribution (i.e., number of offspring) of the available candidates to minimise coancestry between individuals of the current population and the foreign founders. As a result, the candidates with the lowest proportion of exogenous material were favored to produce a higher number of descendants. The strategy proved to be the best method to remove undesired introgression when relying on genealogical information but had some disadvantages, such as increased inbreeding in the population. Moreover, in cases for which the level of introgression was too high or uncontrolled during many generations, the method was relatively inefficient. In a study on the preservation of the genetic background of three cattle breeds, Wellmann et al. [15] also used pedigree information. Although their objective was slightly different, the method constrained exogenous genetic contributions and reduced the probability of identity by descent in the offspring to comparable levels.

In most realistic scenarios, reliable pedigree information is lacking and using molecular information is the only option. In the last decades, molecular markers have become a standard tool to characterize the genetic variation of populations (farmed and natural). Analyses of this molecular variation help to set priorities in conservation programs, to improve traceability in livestock, to estimate genetic diversity among breeds, etc. [16-18]. In a simulation study [19], the information of a few microsatellite-like markers was used to remove undesired introgression through the calculation of genetic distances between the admixed and pure populations or by direct selection of individuals carrying private alleles exclusive to the native population. In all cases, the success was related to the differences in allelic frequencies between exogenous and native populations. In situations in which only a few markers with very similar allele frequencies in both populations were available, the efficiency decreased considerably.

Progress in genotyping techniques has increased the number of available markers and made it possible to use marker information in a wide variety of analyses. The availability of dense molecular marker maps covering the whole genome can provide a more precise picture of the genetic background of a population than pedigree information. Some studies have investigated whether molecular marker information can be a substitute to genealogical information. Hayes et al. [20] demonstrated that replacing the relationship matrix derived from pedigree data with a realised matrix (calculated through genome-wide information) in BLUP analysis, can increase the accuracy of breeding values. De Cara et al. [21] showed that molecular information from high-density markers is more efficient than pedigree data in population management schemes aimed at maintaining genetic diversity using minimum coancestry contributions.

The objective of this study was to analyse, through computer simulations, the consequences of substituting pedigree coancestry with molecular coancestry, calculated from genome-wide marker information, for the removal of exogenous genetic material from an introgressed population.

## Methods

Computer simulations comprised three stages: (1) generation of the two original populations (i.e., native and exogenous); (2) introgression of exogenous individuals into the native population followed by random mating of the resulting offspring for a variable number of generations; and (3) management of the admixed population to recover the genetic background of the native population.

### Native and exogenous populations

Native and exogenous populations included 100 individuals each (50 males and 50 females) and the genome of each individual was made up of 20 chromosomes, each one Morgan long and carrying two types of biallelic loci: 2500 markers and 25 000 non-marker loci. All loci were equidistant and markers were evenly spaced between the non-marker loci.

Initially, frequencies of alleles at each locus (marker and non-marker) were 0.5/0.5 in both populations. To create offspring, random mating was applied in both populations during 100 discrete generations maintaining a constant population size and sex ratio and crossovers were assumed to occur at random along the chromosomes (with no interference) and to follow a Poisson distribution (λ = 1). As a result, allelic frequencies (with a U-shaped distribution due to drift) were divergent between populations and loci were in linkage disequilibrium with a different pattern in each population.

The 2500 markers per chromosome were used in the management of the removal of exogenous background. The non-marker loci were used only for evaluation (not for management), as alleles originating from the native population were distinguishable from those originating from the exogenous population. Therefore, the non-marker loci allowed evaluation of the percentage of genome of each individual that originated from native versus exogenous ancestors, and thus, evaluation of the efficiency of the de-introgression process.

### Exogenous introgression

After generating the native and exogenous base populations, two introgression scenarios were simulated.

#### Scenario involving one introgression event

**Figure 1:**
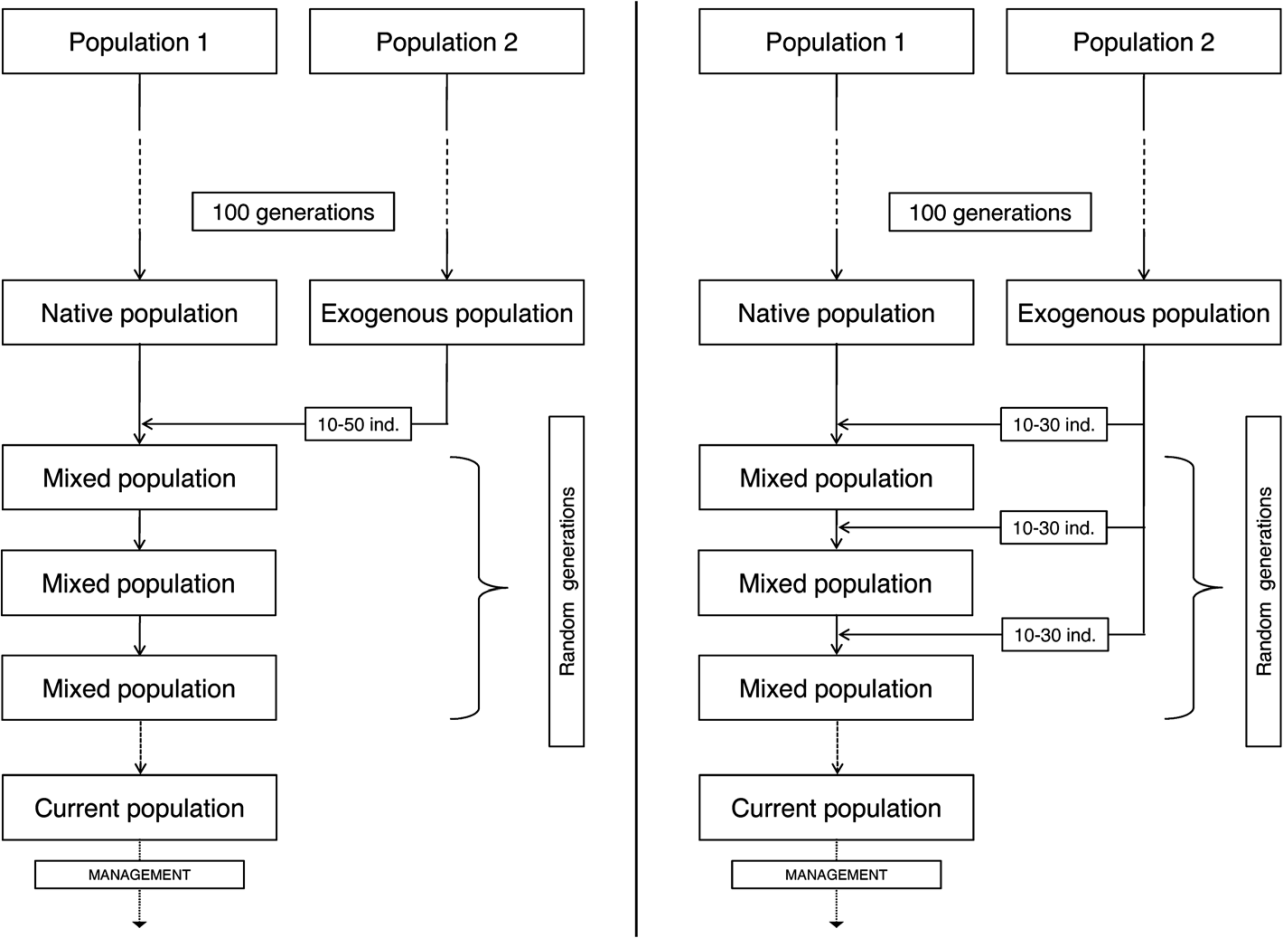
**Design of the two scenarios used in this study.** Left: Scenario involving one introgression event. Right: Scenario involving several introgression events.

To form the admixed population, 10, 20, 30, 40 or 50 exogenous individuals were added to the native population maintaining the total number of individuals at 100 over generations. Individuals were mated randomly during one to five discrete generations (Figure 1).

#### Scenario involving several introgression events

In this scenario, 10, 20 or 30 exogenous individuals were added to the native population at each generation for one to five discrete generations (same number at each generation) without management. The population size was kept constant (*N* = 100) over generations. In generation 1, the non-exogenous individuals originated from the native population and in subsequent generations from the already admixed population (see Figure 1). Equivalences in total percentage of introgression between the two scenarios are shown in Table 1.

**Table 1:** Percentage of introgression in the scenario including several introgression events

	**Number of generations**			
**Number of exogenous individuals per generation**	**2**	**3**	**4**	**5**
10	19.0^**a**^	27.1	34.4	41.0^**d**^
20	36.0	48.8^**c**^	59.0	67.2
30	51.0^**b**^	65.7	76.0	83.2

The table shows the final percentages of introgression in the current population, according to the number of generations of admixture and the number of exogenous individuals per generation, and equivalences with the scenario involving a single introgression event i.e., ^a^20 exogenous individuals entering the population and two generations of admixture, ^b^50 exogenous individuals entering the population and two generations of admixture, ^c^50 exogenous individuals entering the population and three generations of admixture and ^d^40 exogenous individuals entering the population and five generations of admixture.

### Management

After creating the admixed populations, 10 generations of population management were simulated to eliminate the exogenous genetic material. Molecular coancestry based on marker genotypes was calculated between individuals of the current population and the exogenous individuals that were introduced in each generation. A correction was applied to account for marker similarity between individuals in the native and exogenous base populations. Let *g*_*ij*_ be the genotype of individual *i* at SNP *j* with the values "0" if the individual is homozygous for allele 1, "1" if the individual is heterozygous and "2" if the individual is homozygous for allele 2. Then, the standardised genotype *x*_*ij*_ of individual *i* at SNP *j* is [22,23]:

(1) $$x_{\text{ij}}=\frac{g_{\text{ij}}-2p_{j}}{\sqrt{2p_{j}\left(1-p_{j}\right)}}$$

where *p*_*j*_ is the frequency of allele "1" at marker *j* in the base population (i.e., *p*_*j*_ = 0.5). A matrix **X**, composed of *x*_*ij*_ values is constructed for individuals in the current and exogenous populations and the matrix of genomic relationships between current (*c*) and exogenous (*ex*) individuals is calculated as:

(2) $$A=\frac{X_{c}{X'}_{\text{ex}}}{N_{\text{mark}}}$$

where *N*_*mark*_ is the total number of markers. To eliminate exogenous genetic material, at each generation of management, contributions of individuals to the next generation (i.e., number of offspring generated by each potential parent) were calculated by minimising an objective function which includes the relationship between current and exogenous individuals:

(3) $$\sum_{i=1}^{N}c_{i}a_{i,\text{Ex}}$$

where *c*_*i*_ is the relative contribution of individual *i* to the next generation and *a*_*i,Ex*_ is the sum of the genomic relationships between individual *i* and all the exogenous individuals obtained from equation (2). This genomic relationship is equivalent to the molecular coancestry based on identity by state, and standardised. Since calculation of the genomic relationship *a*_*i,Ex*_ is based on many markers, it becomes impossible to have two individuals with the same value. For this reason, minimising the expression in equation (3) selects just one male and one female, i.e., those with the minimum values of *a*_*i,Ex*_. To avoid this, the following restriction was applied: each possible parent in the population could only contribute 10 offspring (of any sex) resulting in 20 equally contributing parents (10 males and 10 females) at each generation, which implies a theoretical rate of inbreeding (*ΔF*) of 0.0125 (assuming random selection and mating). After selection of the 20 parents, they were mated randomly to create the next generation.

To further analyse the implications of the number of individuals contributing to the next generations, a new set of simulations was run, with a maximum number of offspring per individual at each generation of management equal to 5 (resulting in 40 equally contributing parents, theoretical *ΔF* = 0.006). These simulations were run only with the scenario involving a single introgression event.

### Incomplete genotyping of exogenous individuals

Molecular coancestry between individuals in the current and exogenous populations as calculated above considered that genotypes were available for all exogenous individuals. Since this is not always a realistic situation, a new set of simulations was performed in which genotypes were available for only 50% of the exogenous individuals. In this case, management minimised molecular coancestry between individuals in the current population and half of the exogenous individuals that entered the population. Again, these simulations were run only with the scenario involving a single introgression event.

### De-introgression through descendants of the exogenous population

Another set of simulations was performed to investigate a situation in which the individuals that had been used for introgression may not be available but the foreign population from which they originated may be still maintained. A scenario involving a single introgression event but with a percentage of introgression varying between 10 to 50% and five generations of admixture was simulated. In parallel, the foreign population was randomly run for a variable number of generations (the same as in the admixture process). Then, molecular coancestry between individuals of the admixed population and the pure exogenous individuals of this foreign population was calculated and used to remove the exogenous genetic material. In all cases, the number of genotyped descendants and the number of exogenous individuals that originally entered the population were identical.

### Variables used to evaluate the efficiency of the different scenarios

For every generation, two variables were calculated to evaluate the efficiency of the strategies: native genetic representation and average inbreeding coefficient. Native genetic representation is the percentage of native genetic material recovered after one or ten generations of management estimated by the fraction of alleles originating from the native breed based on information from non-marker loci. Native genetic representation relates with the objective of the method itself. Average inbreeding coefficient based on pedigree information reflects the loss of genetic diversity due to the de-introgression process. The rate of inbreeding was calculated for all generations of native and exogenous populations and during admixture and management periods as:

(4) $$\mathrm{\Delta F}=\frac{F_{t}-F_{t-1}}{1-F_{t-1}}$$

The increase in inbreeding accumulated over the ten generations of management is given by *ΔF*_*10*_:

(5) $$\Delta F_{10}=\frac{F_{10}-F_{0}}{1-F_{0}}$$

Twenty replicates per scenario were simulated and the results presented are averages across replicates.

## Results

### Scenario involving one introgression event

#### Native genetic representation

**Figure 2:**
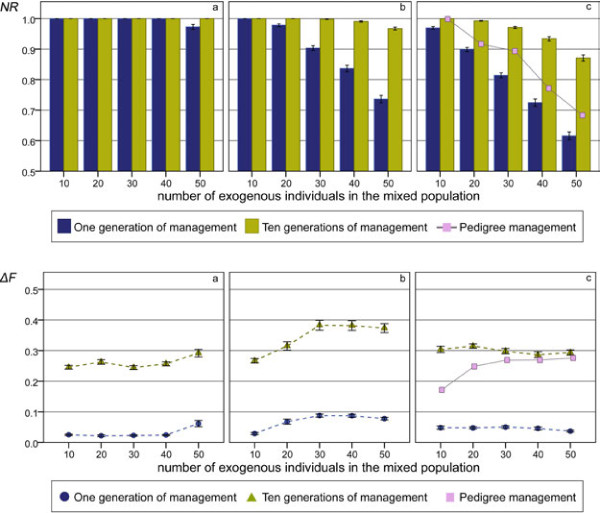
**Native genetic representation and *ΔF *in the simulation with one introgression event.** Native genetic representation is the percentage of native genetic material recovered after one or ten generations of management (upper panels); *ΔF* is average inbreeding coefficient in the first generation and *ΔF*_*10*_ is the increase of inbreeding over ten generations of management (lower panels) in the one introgression event scenarios (maximum number of offspring per individual = 10); (**a**) with one non-managed generation; (**b**) with three non-managed generations; (**c**) with five non-managed generations.

The percentages of native genetic material recovered after one or ten generations of management with the scenario involving a single introgression event (maximum number of offspring per individual = 10) are shown in Figure 2 (upper panel). A notable recovery of native genetic background was obtained by minimising coancestry with the exogenous individuals calculated from information on marker genotypes.

In cases involving just one generation of admixture or minimal introgression, removal of the exogenous genetic material was almost completely achieved in just one generation of management. With increasing percentages of introgression (and/or durations of admixture), more generations of management were required to reach the maximum value.

Compared to the results obtained using pedigree data (only in the scenarios with five generations without management, see Figure 2), the method based on marker data performed markedly better to remove the exogenous genetic material. However, with the pedigree-based method, the maximum level of removal was achieved in just one generation of management [14], thus on this time scale, it is more efficient. With the molecular-based method, an equivalent level of removal to that obtained with the pedigree-based method was achieved only at the second generation of management (not shown), but it continued to increase in subsequent generations. Thus, marker-based management continued to remove exogenous alleles from the population and after 10 generations of management, the overall result was better than with pedigree-based management.

#### Inbreeding coefficient

The observed *ΔF* with the scenario involving a single introgression event (maximum number of offspring per individual = 10) is shown in Figure 2 (lower panel) after one generation of management, as well as the increase in inbreeding accumulated after ten generations of management (*ΔF*_*10*_). The greater increase in *F* compared to expectations for unmanaged populations is a general consequence of the reduction in the number of contributing individuals with the removal method. Notwithstanding, the restriction imposed on the maximum contribution per breeding animal allowed the method to control this increase.

Even when including a restriction on inbreeding, *ΔF* was higher than the theoretical value of 0.0125 in the first generations of management, during which the level of removal was greater. This is due to the fact that the 20 contributing individuals are probably more related to each other than average individuals. This also explains why *ΔF* is higher for scenarios in which recovery of native genetic material is less easily obtained. After a few generations, the maximum removal was almost achieved and the population had more homogeneous coancestry with the exogenous individuals. At this time, observed *ΔF* was close to the theoretical value (data not shown). The more generations the method required to recover the native genetic background, the higher was the total increase in inbreeding.

*F* values obtained with the pedigree-based management and the molecular-based management used here cannot be directly compared. In the pedigree-based method, the algorithm chose solutions with a larger number of contributing parents when several solutions with the same value of global coancestry existed. Therefore, there was an unspecific limitation of inbreeding, but no control over the individuals contributing as in the present study [14]. The performance of pedigree-based management was better (lower *F* values) when introgression was between 10 and 20% and both management systems became similar for medium and high levels of introgression (30 to 50%).

Table 2 shows the percentages of native genetic material recovered and the values of *ΔF*_*10*_ for the scenarios in which the number of offspring per individual in each generation of management was set to 5. The increase in inbreeding was smaller than that obtained in the previous simulations, as expected, which is due to the larger number of contributing individuals. The recovery of native genetic background was lower in most of the scenarios, but exogenous material could still be completely removed if the number of generations of admixture was low.

**Table 2:** **Native genetic representation and*ΔF***_***10***_**in scenarios including five maximum offspring per individual**

	**One generation of admixture**				
**Nb of exogenous individuals per generation**	**10**	**20**	**30**	**40**	**50**
Native genetic representation	1.000	1.000	1.000	1.000	0.999
*ΔF*_*10*_	0.100	0.099	0.113	0.188	0.285
	**Three generations of admixture**				
**Nb of exogenous individuals per generation**	**10**	**20**	**30**	**40**	**50**
Native genetic representation	1.000	0.999	0.984	0.958	0.901
*ΔF*_*10*_	0.116	0.179	0.175	0.175	0.152
	**Five generations of admixture**				
**Nb of exogenous individuals per generation**	**10**	**20**	**30**	**40**	**50**
Native genetic representation	0.997	0.977	0.933	0.873	0.790
*ΔF*_*10*_	0.125	0.127	0.121	0.117	0.110

Values obtained after ten generations of management in the scenario involving one introgression event according to number of exogenous individuals per generation; errors for native representation ranging between 0.000 and 0.012 and for *ΔF*_*10*_ between 0.002 and 0.012.

### Scenario involving several introgression events

#### Native genetic representation

**Figure 3:**
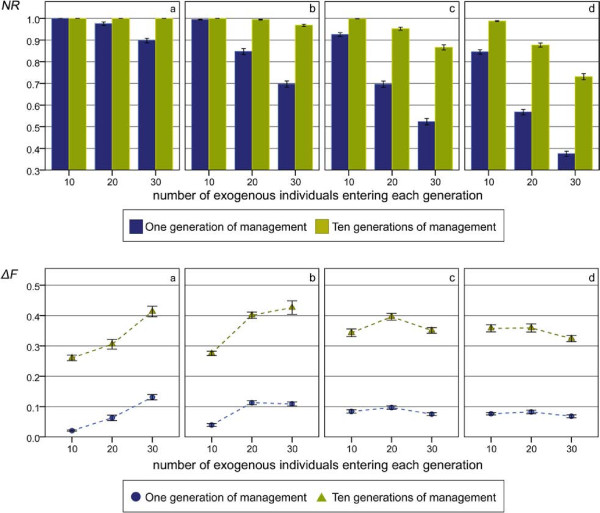
**Native genetic representation and *ΔF *in the simulation with several introgression events.** Native genetic representation is the percentage of native genetic background recovered after one or ten generations of management (upper panels); *ΔF* is average inbreeding coefficient in the first generation and *ΔF*_*10*_ is the increase of inbreeding over ten generations of management (lower panels) in the several introgression scenarios (maximum number of offspring per individual = 10); (**a**) with two non-managed generation; (**b**) with three non-managed generations; (**c**) with four non-managed generations; (**d**) with five non-managed generations.

Figure 3 (upper panel) shows the percentages of native genetic background recovered after one or ten generations of management in the scenario involving several introgression events. In this case too, recovery of native genetic background was substantial and reached 100% in the scenarios with a lower level of introgression.

As observed in the scenario involving one introgression event, native genetic material was recovered to a large extent after one generation of management, especially in cases with a low level of introgression. With higher levels of introgression, more generations were necessary to achieve maximum recovery.

Comparison of equivalent cases between scenarios including one or several introgression events, (i.e., same total percentage of introgression and same number of generations of admixture, see Table 1) showed that, although values are in the same range, the amount of native genetic material recovered was greater with the scenario involving several introgression events than that with a single one.

#### Inbreeding coefficient

As in the previous section, *ΔF* was larger than the theoretical value in the early generations (Figure 3) and decreased to the expected value of 0.0125 in the later generations (not shown). The values of *ΔF* were similar to those obtained in the scenario involving a single introgression event when maximum removal of introgressed genetic material was achieved (Figure 3).

### Incomplete genotyping of exogenous individuals

Table 3 shows the results of the performance of the de-introgression process for the scenario involving a single introgression event and in which only 50% of the exogenous individuals were genotyped. The results show that coancestry between only a few individuals is sufficient to detect exogenous genetic material and to remove it. The recovery level was only slightly lower and involved a lower increase in inbreeding in all cases than when all the genotypes were known.

**Table 3:** **Native genetic representation and*ΔF***_***10***_**in scenarios including a small number of genotyped exogenous individuals**

	**One generation of admixture**				
**Nb of exogenous individuals per generation**	**10**	**20**	**30**	**40**	**50**
**Nb of genotyped exogenous individuals per generation**	**5**	**10**	**15**	**20**	**25**
Native genetic representation	1.000	1.000	1.000	1.000	1.000
*ΔF*_*10*_	0.101	0.101	0.111	0.199	0.292
	**Three generations of admixture**				
**Nb of exogenous individuals per generation**	**10**	**20**	**30**	**40**	**50**
**Nb of genotyped exogenous individuals per generation**	**5**	**10**	**15**	**20**	**25**
Native genetic representation	1.000	0.999	0.987	0.957	0.907
*ΔF*_*10*_	0.116	0.181	0.177	0.178	0.154
	**Five generations of admixture**				
**Nb of exogenous individuals per generation**	**10**	**20**	**30**	**40**	**50**
**Nb of genotyped exogenous individuals per generation**	**5**	**10**	**15**	**20**	**25**
Native genetic representation	0.997	0.973	0.940	0.876	0.808
*ΔF*_*10*_	0.128	0.131	0.130	0.119	0.118

Values obtained after ten generations of management in the scenario involving one introgression event; errors for native genetic representation ranging between 0.000 and 0.013 and for *ΔF*_*10*_ between 0.002 and 0.010.

### De-introgression through descendants of the exogenous population

Table 4 shows the results of the performance of the de-introgression process when using genotypes of descendants of the exogenous population to recover the native genetic background. The level of recovery of native genetic material was slightly smaller in all cases but very similar to that obtained using the original exogenous individuals and the increase in inbreeding was lower due to this loss of efficiency.

**Table 4:** **Native genetic representation and*ΔF***_***10***_**in simulations using genotyped descendants of exogenous individuals**

	**Five generations of admixture**				
**Nb of exogenous individuals per generation**	**10**	**20**	**30**	**40**	**50**
Native genetic representation	0.999	0.992	0.971	0.934	0.871
*ΔF*_*10*_	0.265	0.276	0.269	0.269	0.247

Values obtained after ten generations of management in the scenario including one introgression event after five generations of admixture; *native representation* errors for native genetic representation ranging between 0.000 and 0.013 and for *ΔF*_*10*_ between 0.007 and 0.010.

## Discussion

Benefits of crossbreeding are known and have been widely used in conservation genetics of wild species and livestock [1,2]. However, disadvantages have also been pointed out relating to economic and conservation issues highlighting the benefits of maintaining the purity of some populations [3-5]. Many local breeds have become endangered or extinct because of crossbreeding with more productive breeds [11]. The disappearance of such breeds is potentially detrimental to the genetic basis of livestock production, specifically in the case of particular adaptive characteristics to respond to changes in the environment or market [11,24]. Actions to preserve these breeds are taken worldwide, but if an undesired introgression event happens it will be necessary to recover the original background and develop methods to cope with this situation.

The number of genetic markers available for various livestock and non-farmed species has considerably increased in recent years, reaching 770 000 SNP for cattle. High-throughput genotyping technology has led researchers to reconsider the advantages of using pedigree information versus genotyping data, apart from when pedigree information is completely absent. Genotyping data can successfully replace pedigree information to estimate relationships when marker density is sufficiently high [20,21].

In a previous study, we investigated the efficiency of recovering a native genetic background after introgression by exogenous individuals using a method based on information from a completely recorded pedigree. We showed that small inputs of exogenous alleles can rapidly spread into the population and that it can become very difficult to completely recover the original genetic background. Pedigree information allowed recovery of native genetic background in some situations but at the cost of a high increase in inbreeding [14].

In the present study, we carried out several simulations to test the usefulness of molecular marker-based methods to remove exogenous genetic material. Marker information was used to calculate molecular coancestry that replaced pedigree-based coancestry. Removal of exogenous genetic material present in an admixed population using molecular data was successful, particularly when the level of introgression was low. The marker-based strategy led to a higher de­introgression level than the pedigree-based strategy since it detected more efficiently the exogenous genetic material [14]. Improvements can reach up to 15% (compared to using genealogical information) proving that genome-wide information can be more useful and effective to recover native genetic material after an introgression event.

Similarly to the pedigree-based strategy, the values of *ΔF* obtained with the marker-based strategy showed that each generation of removal increased inbreeding. This suggests that the de­introgression method should be applied for as few generations as possible to avoid the inbreeding effect. In the scenarios with limited introgression, the method required only a few generations to achieve the maximum level of removal of exogenous genetic material. Moreover, the results obtained when the number of offspring is restricted shows that a solution can be found if a high level of *ΔF* cannot be tolerated, while still recovering a large part of the native genome. Before beginning the management process, it is necessary to determine what the acceptable level of increase in inbreeding is, and to apply a restriction on this level to allow for some control on genetic diversity. Once recovery of the native genetic background is completed, the management process should aim at minimising the inbreeding rate using, for example, Optimum Contributions management [25,26].

As Wellmann et al. pointed out [15], one objective could be to recover a large number of native alleles at high frequencies. In this case, individuals with a high percentage of exogenous genes but native alleles not present in the rest of the population should also contribute. Our objective was to completely remove the exogenous genetic material, but genotyping information could also be used to design a breeding program to maximise some specific regions of the genome or combination of alleles. In such cases, a compromise between genetic diversity and removal of exogenous genetic material can be found and implemented.

When the same percentage of exogenous alleles was added to the population progressively (i.e., through several introgression events), the percentage of native genetic material recovered was higher than with the scenario including a single introgression event. This shows that the period of time between when introgression takes place and when removal begins is an important factor. In the scenario involving several introgression events, part of the introgression occurs later than in the scenario with a single introgression event and, thus, the exogenous material is easier to remove.

Other approaches based on marker genotypes have also been shown to be useful to recover an introgressed population [19]. Using information on private alleles led to a substantial recovery of the native background, but required a large number of molecular markers with alleles exclusive to one population, which is not usually the case. Genetic distances were useful when dealing with markers with several alleles, but only in cases for which allelic frequencies were sufficiently different between the native and exogenous populations. Our results are similar or even better (regarding the percentages of native genetic material recovered) than those from studies using private alleles or genetic distances in all comparable scenarios. In addition, if only part of the exogenous individuals is available, they can be used to minimise coancestries and to recover the purest background. Moreover, information on the descendants of the exogenous population instead of the actual exogenous individuals entering the native population can help identify the purest individuals to achieve a high level of recovery of the native genetic material. Again, the efficiency of the removal of exogenous material using partial information would depend on the genetic differentiation between the two admixed populations.

The availability of genome-wide information for natural populations is not yet as widespread as for farmed animals, thus our method is expected to be more easily applied in the latter. However, recent progress in next-generation sequencing approaches makes it possible to identify SNP in species without a reference genome [27,28] thus these techniques should become feasible for wild species in the near future.

Knowledge on the original frequencies of the alleles in the populations is required to correct the genotypes as shown in equation (1). These frequencies should be estimated in the pure population (when still available). If the reference populations are not large, the sampling variance can be corrected as done in other methods to calculate the genomic relationship matrix, e.g., Yang et al. [22].

Besides genealogies and molecular markers, phenotypic characteristics can be used in livestock to characterise a breed. Phenotypes can also be applied to de-introgression as in Fernández et al. [29] and will result in the recovery of certain regions of the genome linked to morphological traits whereas the genome-wide molecular markers provide information all over the genome. Thus, the genetic management approach presented here is flexible and can use alternative sources of information and be applied for different objectives.

## Conclusions

Genome-wide information can be used to remove introgressed genetic material and to completely recover the native genetic background when the contribution of the exogenous population is limited to 30-40% and the number of generations of admixture is not too high (one to three generations). Molecular coancestry proved to be an effective tool to recover native genetic material and even more efficient than pedigree information [14] or information on a small number of markers [19].

Each situation is different and needs to be carefully studied, but even with relatively few SNP, molecular coancestry can be calculated and used in the absence of proper pedigree information for de-introgression. Regardless of the possibilities for recovering a native genetic background, when dealing with introgression it is essential to minimise undesirable exogenous inputs of genetic material as much as possible, because the de-introgression process involves an increased rate of inbreeding that can represent a significant cost for an endangered breed.

## Competing interests

The authors declare that they have no competing interests.

## Authors’ contributions

All authors designed the experiment. CA developed the simulations and methods, and drafted the manuscript. THEM and JFM helped in drafting the manuscript. All authors read and approved the final manuscript.
